# Representation of patients with a migration background in studies on antithrombotic treatment. An analysis of recruitment data from a cluster randomized controlled trial

**DOI:** 10.1371/journal.pone.0230297

**Published:** 2020-03-16

**Authors:** Karola Mergenthal, Andrea Siebenhofer, Lisa-R. Ulrich, Corina Guethlin, Ferdinand M. Gerlach, Juliana J. Petersen

**Affiliations:** 1 Institute of General Practice, Goethe University Frankfurt am Main, Frankfurt am Main, Germany; 2 Institute of General Practice and Evidence-based Health Services Research, Medical University of Graz, Graz, Austria; Inselspital Universitatsspital Bern, SWITZERLAND

## Abstract

**Background:**

The health status, health awareness and health behavior of persons with a migration background often differ from the autochthonous population. Little is known about the proportion of patients with a migration background (PMB) that participate in primary care studies on oral antithrombotic treatment (OAT) in Germany, and whether the quality of their antithrombotic care differs from patients without a migration background. The aim of this paper was to use the results of a cluster-randomized controlled trial (PICANT) to determine the proportion of PMB at different stages of recruitment, and to compare the results in terms of sociodemographic characteristics and antithrombotic treatment.

**Methods:**

This study used screening and baseline data from the PICANT trial on oral anticoagulation management in GP practices. For this analysis, we determined the proportion of PMB during the recruitment period at stage 1 (screening of potentially eligible patients), stage 2 (eligible patients invited to participate in the trial), and stage 3 (assessment of baseline characteristics of patients participating in the PICANT trial). In addition, we compared patients in terms of sociodemographic characteristics and quality of anticoagulant treatment. Statistical analysis comprised descriptive and bivariate analyses.

**Results:**

The proportion of PMB at each recruitment stage declined from 9.1% at stage 1 to 7.9% at stage 2 and 7.3% at stage 3). A lack of German language skills led to the exclusion of half the otherwise eligible PMB. At stages 1 and 3, PMB were younger (stage 1: 70.7 vs. 75.0 years, p<0.001; stage 3: 70.2 vs. 73.5 years, p = 0.013), but did not differ in terms of gender. The quality of their anticoagulant care was comparable (100.0% vs. 99.1% were receiving appropriate OAT, 94.4% vs. 95.7% took phenprocoumon, or warfarin, and the most recent INR measurement of 60.8% vs. 69.3% was within their individual INR range).

**Conclusions:**

In the potentially eligible population and among participants at baseline, the quality of anticoagulant care was high in all groups of patients, which is reassuring. To enable the inclusion of more PMB, future primary care research on OAT in Germany should address how best to overcome language barriers. This will be challenging, particularly because the heterogeneity of PMB means the resulting sample sizes for each specific language group are small.

**Trial registration:**

Current Controlled Trials ISRCTN41847489.

## Introduction

Increasing migration to European countries has resulted in a more diverse population [1]. In 2017, 23.6% of the German population had a migration background. Persons are considered to have such a background when they, or at least one of their parents, did not acquire German citizenship at birth [1].

The health status, health awareness and health behavior of persons with a migration background often differ from the autochthonous population [2]. Furthermore, the proportion of patients with a migration background (PMB) and ethnic minorities participating in epidemiological and health services research studies often diverges from that of the population from which the sample was drawn [3–6]. Studies have shown, for instance, that a lower proportion of PMB participate in studies on cardiovascular disease [3], cancer treatment [7,8] and atrial fibrillation (AF) [9]. Such discrepancies are ethically problematic [10,11] and make it difficult to draw adequate conclusions about health status and effective interventions to improve health [8].

In Germany, most evidence on the recruitment of PMB stems from population-based health surveys (e.g., [12] and other types of epidemiological study (e.g., [13]). Persons with a migration background can be recruited for participation in epidemiological studies through the use of registers (e. g., data from registration offices), based on residential location, via snowball sampling (e. g., through key persons), and via settings [14,15]. Recommended strategies to improve recruitment include, for instance, both proactive (face-to-face) and reactive recruitment strategies (e.g., collaboration with key leaders and printed materials), as well as the employment of ethnically and culturally diverse research staff [15].

However, practical experience varies considerably, depending on the specific contextual factors of the study [14]. Health behaviors, health status and the willingness to participate in a study of those identified using community-based and register-based approaches may differ from samples recruited via primary care providers. Unfortunately, very little is known about the participation and retention rate of PMB in primary care studies in Germany. Over recent years, primary care studies have paid little to no attention to this topic, with most studies simply excluding patients with poor language skills, as is customary in cohort studies conducted in GP practices (e.g., [16]), and cluster-randomized controlled trials on the effectiveness of complex interventions conducted in GP practices (e.g., [17,18]). Thus, little information is available on the proportion of PMB participating in primary care studies, and on whether the health care and health status of such patients differ from those without a migration background.

The success of primary care-based complex interventions depends largely on good communication between GP practice staff and patients, and this is especially true for oral anticoagulation therapy (OAT), which is a challenge for primary care providers, regardless of patient ethnicity [19]. This is because the narrow therapeutic window for optimal doses of coumarins and warfarin complicates the management of oral anticoagulation with these medications [20], while insufficient adherence and a low level of patient knowledge also make OAT difficult to manage [21,22]. Communication problems and language barriers may therefore have a negative impact on the efficacy of OAT.

Between 2012 and 2015, the Institute of General Practice, Goethe University Frankfurt am Main, Germany, carried out the cluster-randomized controlled PICANT (Primary Care Management for Optimized Antithrombotic Treatment) trial, which included 736 patients with a long-term indication for oral anticoagulants in GP practices in Germany [23]. The overall aim was to improve antithrombotic management in primary health care and reduce thromboembolic and major bleeding events by applying major elements of case management.

The aim of this study was to determine the proportion of people with a migration background at different stages of the PICANT recruitment phase (stage 1: screening of potentially eligible patients, stage 2: inviting eligible patients to participate in the trial, stage 3: assessment of baseline characteristics of patients participating in the trial), and to assess whether these results are associated with differences in sociodemographic characteristics and antithrombotic treatment. The specific aims of this analysis were:

1. To determine the proportions of PMB at each of the three stages of the patient recruitment process in the PICANT trial. We defined ‘potentially eligible’ patients as those fulfilling the inclusion criteria for the PICANT trial (> = 18 years, long-term indication for oral anticoagulation, such as atrial fibrillation or recurrent venous thromboembolism, regular attendance of a GP practice). We defined ‘eligible’ patients as those fulfilling the inclusion criteria and not meeting any of the exclusion criteria (e.g., dementia, insufficient German language skills).
2. To assess whether–at stage 1—potentially eligible PMB differed from those without a migration background in terms of sociodemographic characteristics (age, sex) and anticoagulant treatment (type of anticoagulant treatment, self-management of oral anticoagulation, and most recent INR measurement within individual therapeutic range).
3. To assess whether–at stage 3 –PMB that gave their informed consent and participated in the PICANT trial at baseline differed from those without a migration background in terms of sociodemographic characteristics (age, sex) and anticoagulant treatment (type of anticoagulant treatment, self-management of oral anticoagulation, last INR measurement within individual therapeutic range).

This research question was prospectively defined in the published study protocol [23].

## Materials and methods

### Study design and participants

Between June 2012 and March 2015, we conducted the cluster-randomized controlled PICANT trial of patients with a long-term indication for OAT in 52 GP practices in Germany [23]. The trial was registered at Current Controlled Trials (ISRCTN41847489). The institutional review committee of the University Hospital, Goethe University Frankfurt am Main, Germany, approved the study on June 26, 2012 (E 191/11). Details on the study protocol [23] and screening results [24] have been published elsewhere. The aim of the PICANT trial was to assess whether a complex intervention including case management and patient education can improve antithrombotic management in primary healthcare, and reduce major thromboembolic and bleeding events over a follow-up period of 2 years [23]. Practices were eligible for the trial if they provided health care to persons with German statutory health insurance and had a software system at their disposal that was capable of detecting potentially eligible patients. Practices already participating in studies aimed at improving the quality of oral anticoagulation were excluded.

Recruitment of practices and patients took place between June 2012 and December 2012. We identified potentially eligible practices from a list provided by the Association of Statutory Health Insurance Physicians (mandatory registration of GP practices). As the list only contains the names and addresses of GPs, we mailed information on the trial to 568 randomly selected practices (6% of all registered practices in 2012) and invited them to participate. Inclusion criteria were only checked for those that were interested in participation. Practice recruitment was stopped when 52 practices had enrolled, even though further practices were interested in taking part. Each participating practice was visited after practice recruitment, but before cluster randomization.

Patients were eligible for inclusion in the main trial if they were ≥ 18 years of age, had a long-term indication for oral anticoagulation based on the guidelines valid at the time, and were prescribed coumarins, antiplatelet therapies, or the DOACs that were on the market when the study began (dabigatran, rivaroxaban). Exclusion criteria were dementia, diseases resulting in a life expectancy of < 6 months, psychosis, severe sight disorders or auditory defects, alcohol- or drug abuse, residence in institutions that did not allow study participation, and a lack of German language skills.

To identify potentially eligible patients, we asked practice teams to use their practice software to generate a pseudonymized screening list based on predefined instructions and search terms provided by the study team members [23]. The GPs then checked the lists and deleted eventual cases of patients that had only been seen occasionally, or had died in the meantime. Inclusion criteria were then assessed by the GP for randomly selected patients from the list. To avoid selection bias, the order of the patients assessed for eligibility was chosen by means of the random number generator function in Microsoft Excel®. This screening process was performed in each practice until 30 potentially eligible patients had been identified. These 30 patients received a written invitation to participate in the trial from their GP. Patient recruitment was stopped after 15 patients had provided informed consent to participate and baseline data. After the baseline assessment had been completed, a member of the Institute of General Practice that had no further involvement in the study used the web-based randomization tool “Randomizer for Clinical Trials” (www.randomizer.at) to consecutively and randomly allocate practices to the intervention or routine care arm in a ratio of 1:1. Randomization was stratified according to the number of inhabitants in the postal area where the practice was located and using permuted blocks of size 8. The statisticians were blinded to group assignment during the analysis [24].

We used written consent procedures, and all participants (practice teams, patients) in the PICANT trial gave their informed consent.

### Data collection

In the main PICANT trial, data collection consisted of three assessments (at baseline, after 12 and 24 months) using self-rating questionnaires for patients, and case report forms for GPs. Furthermore, during the screening process, study team members interviewed the GP practice teams and filled in a pseudonymized documentation sheet for each screened patient (only the practice teams knew the patient’s name). The documentation sheet included information on patient age, sex, long-term indication for OAT, antithrombotic medication, whether patients performed self-management, reasons for exclusion, most recent INR value, as well as information on migration background and, when appropriate, German language skills (assessed by the practice teams and ranging from 1 ‘excellent’ to 6 ‘insufficient). Each screened patient was then allocated to one of the following three categories: Category 1: patients with a long-term indication for OAT and taking anticoagulants (i.e., patients receiving ‘appropriate’ OAT therapy); category 2: patients with a long-term indication for OAC but not taking anticoagulants (i.e., ‘under-treated’ patients, for whatever reason); category 3: patients without a long-term indication for OAT but taking an anticoagulant on a permanent basis (i.e., ‘over-treated’ patients, for whatever reason. Only patients from categories 1 and 2 were eligible for study participation (S1 File).

During the screening process, the practice teams filled in a further documentation sheet for each of the 30 eligible patients invited to participate, in order to assess whether patients had agreed to participate. Reasons for non-participation were also documented.

### Statistical analyses

For the descriptive analyses, we calculated mean value, standard deviation and the frequency distributions of the response categories. For bivariate analyses, we performed Chi-Square, t-, and Mann-Whitney tests. All p values were 2-sided and considered statistically significant at a significance level of < 0.05. We used IBM SPSS (Statistical Package for Social Sciences) Statistics for Windows, version 22.0, Armonk, NY: IBM Corp [25] for the remaining analyses.

## Results

**Fig 1:**
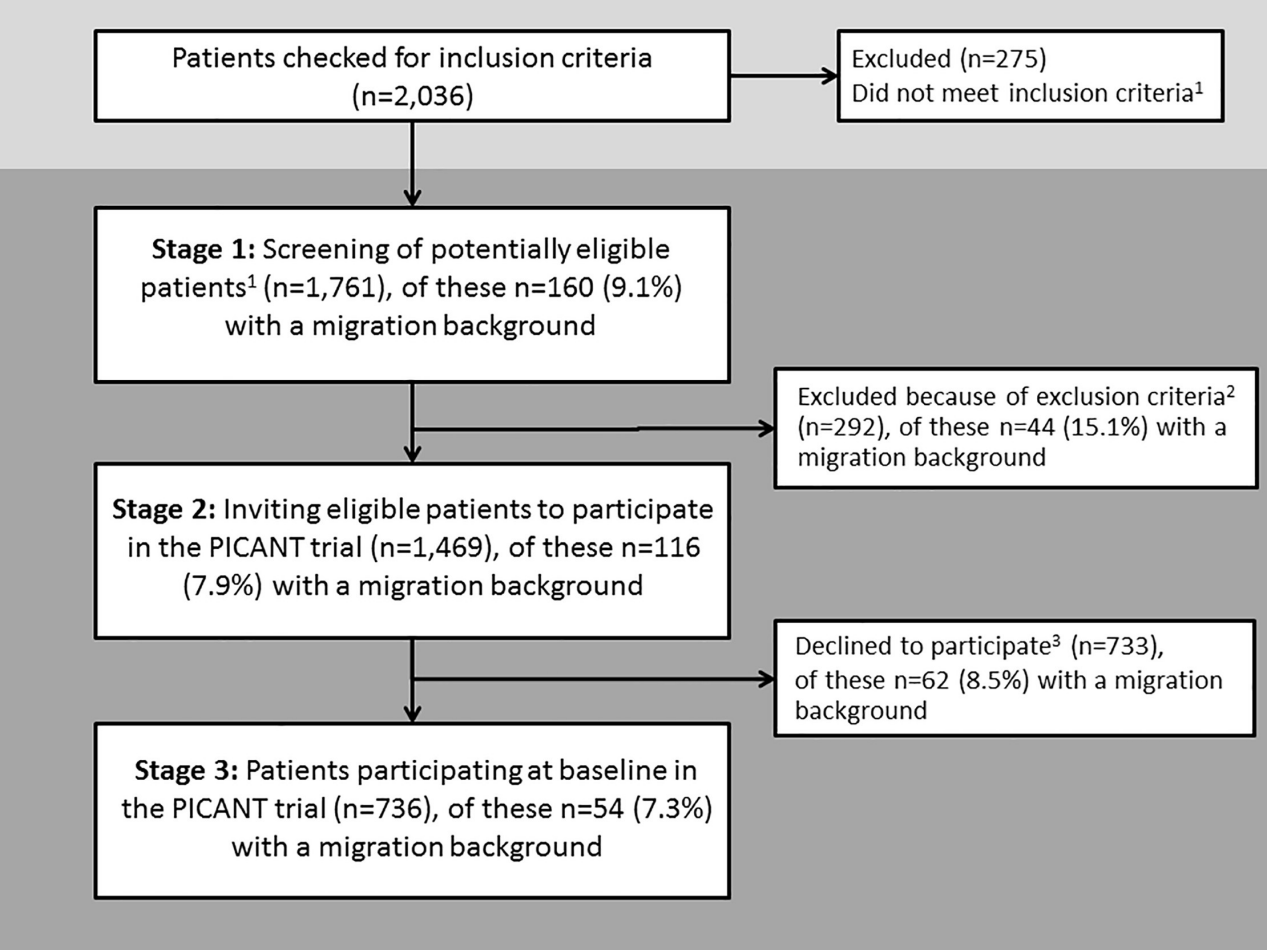
Proportion of patients with a migration background at all stages of the recruitment process. ^1^ ≥18 years of age; long-term indication for oral anticoagulation (atrial fibrillation/flutter, recurrent venous thromboembolism or pulmonary embolism, mechanical heart prosthesis and others, such as hereditary coagulopathy, intracardial thrombosis) and an indication for coumarins, antiplatelet therapies, or the new direct antithrombotic agents rivaroxaban and dabigatran; regularly attend the GP’s practice ^2^ Dementia; disease resulting in a life expectancy of less than six months; psychosis; severe sight disorders or auditory defects; alcohol or drug abuse; residence in institutions (e.g. nursing homes or residential care homes), and a lack of German language skills as assessed by the GP ^3^ Failed to get in touch within four weeks; no interest; a lack of German language skills; interested, but target of 15 participants already achieved; patient felt too ill or too old; time constraints; other reasons for non-participation; temporary change in home address or moved away.

We included 52 GP practices, and screened 2,036 patients for eligibility. Of these, 1,761 fulfilled the inclusion criteria and were therefore classified as being ‘potentially eligible’ (stage 1; Fig 1). Of the 1,761 patients, 160 (9.1%) had a migration background. These patients came from 29 different countries. The six most frequent countries of origin were: the Czech Republic (n = 19, 12%), Italy (n = 17, 11%), Poland (n = 13, 8%), former Yugoslavia (n = 11, 7%), Russia and Turkey (each: n = 6, 6%). The proportion of patients with a migration background fell at each subsequent recruitment stage, i.e. from 9.1% at stage 1 to 7.9% at stage 2 (eligible patients invited to participate in the PICANT trial), and 7.3% at stage 3 (patients actually participating in the PICANT trial) (Fig 1).

### Reasons for study exclusion

An analysis of the criteria leading to study exclusion indicated that most patients (38.0%) were excluded because of dementia (Table 1). However, when the excluded patients were analyzed in terms of migration status, it became apparent that nearly half (45.4%) the exclusions of PMB from study participation were because their GPs considered them to have insufficient German language skills.

**Table 1:** Descriptive analysis of criteria assessed in patients with and without a migration background that led to study exclusion^1^.

	all patients meeting exclusion criteria, n (%)	with a migration background, n (%)	without a migration background, n (%)
Dementia	111 (38.0)	7 (15.9)	104 (41.9)
No long-term indication for oral anticoagulation	42 (14.4)	7 (15.9)	35 (14.1)
Life expectancy < 6 months	21 (7.2)	1 (2.3)	20 (8.1)
Lack of German language skills	20 (6.8)	20 (45.4)	0
Residence in nursing home or residential care home	14 (4.8)	0	14 (5.6)
Severe sight disorder or auditory defect	9 (3.1)	1 (2.3)	8 (3.2)
Alcohol or drug abuse	5 (1.7)	0	5 (2.0)
Psychosis	5 (1.7)	1 (2.3)	4 (1.6)
Other reasons for exclusion	65 (22.3)	7 (15.9)	58 (23.4)
Total	**292 (100%)**	44 (100%)	248 (100%)

^1^ As assessed by GP

### Reasons for non-participation of eligible patients

The analysis of reasons for the non-participation of eligible patients invited to participate in the trial showed that 12 (20%) non-participants with a migration background gave "lack of German language skills" as the reason (Table 2).

**Table 2:** Description of reasons for non-participation of eligible patients with and without a migration background^1^.

	all eligible patients invited to participate in the main trial, n (%)	with a migration background, n (%)	without a migration background, n (%)
Patient did not response within predetermined 4-week period	341 (46.5)	29 (46.7)	312 (46.5)
Patient was not interested in participation^1^	217 (29.6)	9 (14.5)	208 (31.0)
Patient reported lack of German language skills^1^	12 (1.6)	12 (19.4)	0
Patient was interested in participation, but excluded because recruitment target for the corresponding GP practice (15 participants) had already been achieved	57 (7.8)	3 (4.9)	54 (8.0)
Patient felt too sick or old for participation^1^	54 (7.4)	4 (6.5)	50 (7.5)
Patient reported time constraints^1^	25 (3.4)	2 (3.2)	23 (3.4)
Other reasons for non-participation^1^	19 (2.6)	2 (3.2)	17 (2.5)
Patient had moved away	5 (0.7)	0	5 (0.7)
Missing data	3 (0.4)	1 (1.6)	2 (0.3)
**Total**	**733 (100%)**	62 (100%)	671 (100%)

^1^ As documented by GP practice staff (after asking the patient)

### Comparison of patients with and without a migration background

At stage 1, potentially eligible patients with a migration background were younger than those without a migration background (70.7 vs. 75.0 years; p <0.001; Table 3). The two groups did not differ statistically significantly with regard to sex and anticoagulant treatment: 94.8% vs. 95.5% received appropriate OAT (p = 0.70), 93.3% vs. 93.6% took phenprocoumon or warfarin (p = 0.86), 10.5% vs. 9.5% (p = 0.46) self-managed their oral anticoagulation, and the most recent INR measurement of 63.0% vs. 67.1% (p = 0.33) was within the individual’s therapeutic range.

**Table 3:** Comparison of sociodemographic characteristics and anticoagulation treatment of potentially eligible patients and participants with a migration background with those without a migration background.

	Potentially eligible patients (i.e., meeting inclusion criteria; n = 1,761)^1^			Patients participating at baseline of the PICANT trial (n = 736)		
Results	with a migration background (n = 160)	without a migration background (n = 1,597)	p-value	with a migration background (n = 54)	without a migration background (n = 682)	p-value
Mean age in years (SD)	70.7 (10.7)	75.0 (10.2)	**<0.001**^2^	70.2 (8.67)	73.5 (9.47)	**0.013**^2^
Male sex (n, %)	77 (52.7)	847 (53.0)	0.236^3^	25 (46.3)	380 (55.7)	0.180^3^
Appropriate OAC therapy (n, %)						
Patients with long-term indication for OAC and receiving anticoagulant treatment	147 (94.8)	1,491 (95.5)	0.700^3^	54 (100.0)	676 (99.1)	1.000^3^
Antithrombotic medication, n %)						
*phenprocoumon (e*.*g*., *Marcumar®) or Coumadin®*	140 (93.3)	1,412 (93.6)	0.862^3^	51 (94.4)	645 (95.7)	0.666^3^
*dabigatran (Pradaxa®) or rivaroxaban (Xarelto®)*	10 (6.7)	97 (6.4)		3 (5.6)	29 (4.3)	
Last INR measurement within individual therapeutic INR target range, n (%)	87 (63.0)	917 (67.1)	0.331^3^	31 (60.8)	446 (69.3)	0.209^3^
INR self-measuring and dose adjustment, n (%)	15 (10.5)	135 (9.5)	0.455^3^	10 (19.6)	76 (11.8)	0.102^3^

^1^ n = 1,765 patients participated in the assessment. Of these, n = 4 were excluded from these analyses because GPs did not provide sufficient data

^2^ t-test

^3^ Chi^2^-test (Pearson)

At stage 3, PMB that participated in the baseline assessment of the PICANT trial were younger (70.2 vs. 73.5 years; p = 0.013) and less frequently female (46.3% vs. 55.7%; p = 0.18) than patients without a migration background. Overall, 100.0% vs. 99.1% received appropriate OAT (p = 1.0) and the groups received similar anticoagulant treatment, with 94.4% vs. 95.7% (p = 0.66) taking phenprocoumon or warfarin, 19.6% vs. 11.8% (p = 0.10) self-managing their oral anticoagulation, and the last INR measurement of 60.8% vs. 69.3% (p = 0.21) being within the individual’s therapeutic range. Of the PMB, 87.0% (140 of 160) had sufficient German language skills according to the information provided by the GP.

## Discussion

This study provides new findings and insights into the extent to which PMB are represented at different stages of the recruitment phase of a large cluster-randomized controlled trial in Germany. During the recruitment process for this trial, the proportion of migrants decreased slightly at each stage. It shows that in 2012 the proportion of PMB among adults with a long-term indication for oral anticoagulation and treated regularly in GP practices was about 9%. In the same year, PMB made up more than 23% of the population as a whole [26]. The proportions probably differed because in Germany, the percentage of migrants is lower among elderly persons than in the overall population, and the average age of our sample was relatively high.

A lack of German language skills was an exclusion criterion in the PICANT trial and was responsible for almost half the exclusions of PMB that would otherwise have been eligible for inclusion. This is in line with the findings of Gill et al. [2], who identified language barriers, and an inability to understand what it means to participate in a research project, as two of the main reasons why ethnic minorities did not participate in a cardiovascular research study in the UK [2]. Although often cited and also used as an exclusion criterion in the PICANT trial for reasons of practicality and cost, an inability to speak and understand the native language should not be a general exclusion criterion [27]. Future primary care research in Germany should address how best to overcome these barriers (e.g. specific recruitment strategies, use of interpreters or translation of questionnaires) [4]. As can be seen from our results, the heterogeneity of PMB and the small numbers of patients speaking specific languages will make this a challenge. Furthermore, the specifics of primary care research (embedded in a busy, everyday care setting) make it difficult to employ strategies that might work in a more structured setting, such as the use of interpreters. However, increasing numbers will make the adequate representation of PMB increasingly relevant in Germany in the future [1].

A comparison of the baseline characteristics of potentially eligible patients and the participants in this trial showed that, on average, patients with a migration background were considerably younger than those without. The difference probably reflects the younger average age of PMB in the German population as a whole [1]. We therefore performed an exploratory analysis (using a Cox proportional hazards model with robust sandwich estimates to account for clustering) to investigate whether migration background had any influence on the incidence of the primary outcome when age and randomization group were considered to be confounders. We did not observe a significant effect (HR 0.89, 95% confidence interval 0.38–2.09, p = 0.794), which may reflect the small number of PMB.

In the potentially eligible population and among participants at baseline, the quality of anticoagulant care was high in all groups. This result is reassuring, since previous research has indicated that PMB receive poorer care than those without. The generally high quality of OAT care in our study confirms the findings of a previous study that took place in a German general practice setting [28].

### Strengths and limitations

To the best of our knowledge, this is the first study in primary care research in Germany to assess the proportion of patients with a migration background at different stages of the recruitment process of a large cluster-randomized controlled trial in GP practices. However, the study has several limitations. Data collection at stages 1 and 2 was mainly based on information derived from practice teams, so information on a patient’s migration background and German language skills was not self-assessed in these cases. Obtaining informed consent from patients while they are being screened for eligibility is not feasible in this setting. However, since patients had a long-term indication for oral anticoagulation and were regularly monitored in the practice, we assume that patients were well known by the practice teams (In Germany, GP practices are usually small and privately owned, and one or two GPs work in them). A further limitation of this study is that we present the results of a cross-sectional study in general practices without any stratification of the German migrant population. However, we randomly selected GPs and patients according to good clinical practice guidelines [23]. Because of the study design, we could only carry out descriptive analyses. It was therefore impossible to make causality assumptions, and we were unable to assess, for instance, the extent to which language skills may have influenced the quality of anticoagulation. We did not carry out a sample size calculation for this study because our sample size calculation was performed for the primary outcome of the main trial [23]. However, this study was preconceived and described in the study protocol before data collection.

## Conclusion

In the potentially eligible population and among participants at baseline, the quality of anticoagulant care was high in all groups, which is reassuring. However, to enable the inclusion of more PMB, future primary care research on OAT in Germany should address how best to overcome language barriers. This will be challenging, particularly because the heterogeneity of PMB means the resulting sample sizes for each specific language group are small.

## Supporting information

S1 File (PDF)
