# Supplementary material for: Representation of patients with a migration background in studies on antithrombotic treatment. An analysis of recruitment data from a cluster randomized controlled trial
Source: PLoS One. 2020 Mar 16;15(3):e0230297. doi: 10.1371/journal.pone.0230297 (PMC7075549; doi:10.1371/journal.pone.0230297)
Supplement: S1 File — (PDF) [file pone.0230297.s001.pdf]

Picant
Primary Care Management for Optimized Antithrombotic Treatment

Screening-ID ☐☐ - S ☐☐

Screening sheet

Date ☐☐ ☐☐ 2012
T T M M

1. Gender of patient: ☐ Female ☐ Male

2. Patient's month and year of birth: ☐☐ (M M) ☐☐ (J J)

3. Which of the following indications for an oral anticoagulant are present in the patient?
(Multiple answers possible; please give family doctor response card 1)

☐ **Chronic atrial fibrillation/flutter** (paroxysmal, persistent or permanent)

If so, please also provide CHA_2DS_2VASc score: (give family doctor response card 2)

☐ CHA_2DS_2-VASc score > 1

☐ CHA_2DS_2-VASc score = 1

☐ CHA_2DS_2-VASc score = 0

☐ **Deep vein thrombosis (DVT)**

If so, please specify:

☐ High risk of recurrence: repeated episodes of, or current severe thrombophilia

☐ Low risk of recurrence: first episode provoked by transient (reversible) risk factor or primary idiopathic thrombosis

☐ **Left atrial enlargement (LAE)**

If so, please specify:

☐ High risk of recurrence: repeated episodes of, or current severe thrombophilia

☐ Low risk of recurrence: first episode provoked by transient (reversible) risk factor or primary idiopathic thrombosis

☐ **Mechanical heart valve**

☐ **Intracardiac thrombi**

☐ **Other indication, namely**: ______________________________

4. What <u>permanent</u> anticoagulant drug(s) is the patient taking? *(please give family doctor response card 3)*

☐ phenprocoumon (e.g. Marcumar®) ☐ dabigatran (Pradaxa®)

☐ rivaroxaban (Xarelto®) ☐ acetylsalicylic acid (e.g. Aspirin®)

☐ another blood-thinning medication: ______________________________

☐ no medication because:

____

5. Which study group will the patient be allocated to? *Please use the "flowchart to determine list of patients" and allocate patient to only <u>one</u> group!*

**Group 1:** A long-term oral anticoagulant (OAC) is indicated <u>and</u> patient is taking one	☐	***→ continue to question 6***
**Group 2:** A long-term OAC is indicated but patient is not taking one	☐	***→ continue to question 6***
**Group 3:** A long-term anticoagulant is <u>not</u> indicated, but patient is currently taking one	☐	***→ Enter into "Group 3 list of patients" and continue to question 7***
**Other reasons** (e.g. patient is receiving a one-off OAC therapy because episode occurred 3-6 months previously, or is receiving an anticoagulant due to an arterial event)	☐	***→ Questionnaire ends here***

6. Do any of the following exclusion criteria apply to the patient? *(Multiple answers possible; give family doctor response card 4; If the patient is to participate in the intervention, the doctor should assess whether regular contact with the HCA will be possible)*

☐ Dementia that in the doctor's opinion would prevent participation in the study

☐ Final stage of the disease (estimated life expectancy < 6 months)

☐ Psychosis

☐ Severe visual or auditory impairment that in the doctor's opinion would prevent participation in the study

☐ Alcohol or drug abuse that in the doctor's opinion would prevent participation in the study

☐ Accommodation in a facility (e.g. nursing or old people's home) that in the doctor's opinion would prevent participation in the study

☐ Inadequate command of the German language

☐ Other exclusion criterion, namely: ______________________________

☐ **There are no reasons for exclusion →** *please enter patient into the list and allocate him or her a new consecutive ID:*

Allocated patient ID: ☐☐-P ☐☐

7. What is the patient's therapeutic INR target range?

INR ☐.☐ **to** ☐.☐

8. Most recent INR value: ☐.☐ **Date of measurement:** ☐☐ (M M) 20 ☐☐ (J J)

9. Does the patient measure INR value and adjust the dose independently?

☐ No

☐ Yes, measures INR and adjusts dose independently

☐ Yes, measures the INR value but does not adjust the dose independently

If the patient performs self-management:

9.1 On whose initiative did the patient undergo self-management training?

____

9.2 Which training center did the patient go to?

____

10. How compliant is the patient in the doctor's opinion?

☐ very compliant ☐ moderately compliant ☐ not compliant

Any other comments/ remarks:________________________________

11. Does the patient have a migrant background?

A migrant background is considered to exist if the patient does not have German citizenship, and/or was not born in Germany, and/or one or both of the patient's parents were born outside Germany.

☐ Yes → ***continue with question 12***

☐ No → ***Questionnaire ends here***

12. Patient's country of birth:

☐ Germany

☐ Another country of birth: ______________________________

☐ Country of birth is unkown

13. Patient's native language:

☐ German

☐ Another language: ______________________________

☐ Native language is unknown

14. How would you assess the patient's command of the German language?

1 ☐=very good 2 ☐=good 3 ☐=satisfactory

4 ☐=adequate 5 ☐=poor 6 ☐=inadequate
